# Effect of spatial origin and hydrocarbon composition on bacterial consortia community structure and hydrocarbon biodegradation rates

**DOI:** 10.1093/femsec/fiy127

**Published:** 2018-06-30

**Authors:** Lloyd D Potts, Luis J Perez Calderon, Evangelia Gontikaki, Lehanne Keith, Cécile Gubry-Rangin, James A Anderson, Ursula Witte

**Affiliations:** 1 Institute of Biological and Environmental Sciences, School of Biological Sciences, University of Aberdeen, Cruickshank Building, St Machar Drive, Aberdeen, AB24 3UL, United Kingdom; 2 Materials and Chemical Engineering, School of Engineering, University of Aberdeen, Fraser Noble Building, Elphinstone Road, Aberdeen, AB24 3UE, United Kingdom

**Keywords:** Faroe-Shetland Channel, Hydrocarbon Degradation, Microbial Community, Bacteria, Deep-sea

## Abstract

Oil reserves in deep-sea sediments are currently subject to intense exploration, with associated risks of oil spills. Previous research suggests that microbial communities from deep-sea sediment (>1000m) can degrade hydrocarbons (HCs), but have a lower degradation ability than shallow (<200m) communities, probably due to *in situ* temperature. This study aimed to assess the effect of marine origin on microbial HC degradation potential while separating the influence of temperature, and to characterise associated HC-degrading bacterial communities. Microbial communities from 135 and 1000 m deep sediments were selectively enriched on crude oil at *in situ* temperatures and both consortia were subsequently incubated for 42 days at 20°C with two HC mixtures: diesel fuel or model oil. Significant HC biodegradation occurred rapidly in the presence of both consortia, especially of low molecular weight HCs and was concomitant with microbial community changes. Further, oil degradation was higher with the shallow consortium than with the deep one. Dominant HC-degrading bacteria differed based on both spatial origin of the consortia and supplemented HC types. This study provides evidence for influence of sediment spatial origin and HC composition on the selection and activity of marine HC-degrading bacterial communities and is relevant for future bioremediationdevelopments.

## Teaser

This study describes the effect of seabed sediment origin on microbial hydrocarbon degradation rates and the influence of different HC mixtures on bacterial consortia composition.

## INTRODUCTION

Continued demand for petroleum is forcing oil exploration into deeper waters such as the United Kingdom’s continental slope west of the Shetland Islands (Austin, Cannon and Ellis 2014). Here, any potential oil release would be difficult to contain, monitor and remediate considering the challenging environment deep-sea areas pose (Cordes *et al.*^2016^). This was evidenced following the Deepwater Horizon (DWH) deep-sea well blowout in the Gulf of Mexico (GoM) at a depth of ∼1500 m (DWH Natural Resource Damage Assessment Trustees 2016), which resulted in the formation of deep-sea oil intrusion layers (Camilli *et al.*^2010^). Post-spill, it was established that between 3% and 31% (Chanton *et al.*^2015^; Valentine *et al.*^2014^) of the ∼4.9 million barrels of spilt oil were transported to the seabed (Brooks *et al.*^2015^; Romero *et al.*^2015^). Investigations into the impact of oil deposition on deep-sea sediments revealed the presence of bacteria known to be capable of hydrocarbon degradation, although the *in situ* function was not determined (Kimes *et al.*^2013^; Mason *et al.*2014b). Within certain regions of the marine environment, frequent oil influx from naturally occurring seeps has allowed local microbial communities to adapt to and exploit the carbon-rich substrate as an energy source (LaMontagne *et al.*^2004^). This results in a ‘primed’ ecosystem that has an intrinsic capacity to respond to and degrade HCs (Hazen *et al.*^2010^). It is believed this was in part responsible for the rapid response of HC degraders in the water column and benthos following DWH (Hazen *et al.*^2010^). In areas lacking natural seeps, in the event of an oil spill, microbial communities may not be as responsive and further investigation is required to determine the ability of such sites to naturally remediate oil pollution.

HC-degrading microbial community assembly is largely dependent upon environmental conditions (Coulon *et al.*^2007^; Duran and Cravo-Laureau 2016). Temperature and hydrostatic pressure (among other environmental parameters) select for distinct site-specific communities that can in turn influence intrinsic degradation ability. Microbial community analysis of oil contaminated samples at cold temperatures (−5°C to 20°C) often reveals the dominance of psychrophilic and psychrotolerant organisms such as *Colwellia* (Methé *et al.*^2005^; Mason *et al.*2014a) and *Pseudomonas* (Malavenda *et al.*^2015^), respectively. Hydrostatic pressure increases with water column depth and is also known to alter community composition (Fasca *et al.*^2017^; Marietou *et al.*^2018^). For example, following DWH, more heterotrophic HC degraders than hydrocarbonoclastic bacteria were detected (Scoma, Yakimov and Boon 2016), a result that contrasts with findings from surface water analysis (Kimes *et al.*^2014^). Indeed, HC degradation can be regulated by environmental parameters such as temperature and hydrostatic pressure (Schwarz, Walker and Colwell 1974; Bagi *et al.*^2014^), however, degradation rates are also probably dependent on site-specific composition of microbial communities.

HCs are thought to be degraded in a predictable manner based on their structural complexity. Typically this follows an order of: low molecular weight aliphatic (C_6_–C_20_) and aromatic (1–2 rings) HCs, followed by simple branched alkanes, before degradation of long chained (C_20+_) alkanes and heavy molecular weight polyaromatic hydrocarbons (PAHs), and finally resins and other polar fractions (Head, Jones and Röling 2006). Organisms preferentially utilise certain structures of HCs, sometimes irrespective of typical degradation models. *Alcanivorax* (Yakimov and Golyshin 1998) and *Oleispira* (Yakimov 2003) utilise a variety of branched and/or straight chain aliphatic HCs, whereas *Cycloclasticus* (Dyksterhouse *et al.*^1995^) and *Marinobacter* (Vila *et al.*^2010^) are known to degrade PAHs. Different mixtures of HCs promote differential responses in complex microbial communities whereby particular microbes may dominate or be outcompeted (McKew *et al.*^2007^). This highlights preferential degradation and competition within communities that may challenge expected degradation kinetics.

The Faroe–Shetland Channel (FSC), located in the North East Atlantic, north of the United Kingdom, harbours oil reserves in waters >1000m deep and has been subject to increased well drilling and production. By contrast to the GoM, the FSC presents an environment where there is no evidence to suggest microbes are predisposed to HC degradation through natural seepage. Temperatures in the deep FSC are ∼0°C that may structure communities differently to shallower, warmer, regions and reduce HC degradation rates as previously evidenced (Ferguson *et al.*^2017^). The FSC displays a unique, complex and dynamic hydrographic regime (Berx *et al.*^2013^) that would likely result in oil being carried much farther afield than witnessed during the DWH disaster (Main *et al.*^2017^). It is therefore required to gain a better understanding of the biodegradation capabilities of microbial populations indigenous to the FSC.

To further knowledge of the maximal HC degradation capabilities and microbial community responses in the FSC, this study aimed to evaluate the ability of FSC seabed bacterial communities from two different depths (shallow, 135 m and deep, 1000 m) to degrade a range of HCs while removing the effect of temperature. Due to changes in conditions between the two depths such as temperature and hydrostatic pressure, which drive community differentiation, it was hypothesised that the two enriched consortia would have had different HC degradation capabilities when incubated at the same temperature and that the shallower community would be capable of increased degradation (Ferguson *et al.*^2017^). Furthermore, different HC profiles would influence bacterial community composition as differences in HC structure can select for different taxa (McKew *et al.*^2007^). The primary aims of this study were, (1) to assess the influence of site-specific community composition on HC degradation rates, (2) to determine the differential responses of consortia when exposed to two different mixtures of HCs over time and (3) to monitor temporal changes in bacterial community structure induced by preferential HC degradation. In addition, this study aimed to isolate HC-degrading bacteria to increase understanding of HC utilisation and surfactant production capability.

## MATERIALS AND METHODS

### Sampling of marine sediments and enrichment of crude oil degrading consortia

Sediments were obtained from two stations within the FSC. Shallow sediments sampled at 135 m (FSC135, hereafter) were provided by Premier Oil Ltd in November 2014. Deep sediments were sampled at 1000 m (FSC1000, hereafter) using a day grab on the *MRV Scotia* (cruise number Sc201405) between 24/04/14–08/05/14. Detailed sediment sampling information is provided in Table S1 (Supporting Information). To select for HC-degrading bacteria, 1 g of collected seabed sediments (FSC135 or FSC1000 separately) were added into 100 mL Erlenmeyer flasks containing 30 mL sterile ONR7a medium (a mineral salts medium made up according to Dyksterhouse *et al*. 1995) and 1% vol/vol autoclaved crude oil (Schiehallion crude oil, provided by BP plc; formerly British Petroleum) as the sole carbon source. It is noted that only the water accommodated fraction and some mechanically dispersed oil droplets will reach the sediment and thus impact microbial communities, while the remainder floats on the medium surface. Flasks were set up in triplicate and agitated at 150 RPM for 2 weeks at *in situ* temperature (12 ± 2°C and 0 ± 2°C for FSC135 and FSC1000, respectively) in the dark. Following incubation, 3 mL of enriched culture was transferred to fresh ONR7a/crude oil medium, and this procedure was repeated thrice more. At the end of this enrichment procedure, replicate samples were pooled and stored at −80°C in sterilised glycerol (20% final concentration). Glycerol cryopreservation may selectively preserve certain taxa based upon the ability of glycerol cell wall penetration that varies between taxa (Prakash, Nimonkar and Shouche 2013), although a recent study demonstrated consistent preservation of dominant microbes (Yu *et al.*^2015^). It must be noted that since molecular analysis was not performed on sediments before enrichment, the composition and structure of pre-exposed communities is not provided; however, it is expected to be similar to analyses from previous studies from the same location (Ferguson *et al.*^2017^; Perez Calderon *et al.*^2018^).

### Microcosm setup and sampling strategy

Two different HC mixtures were used to evaluate their respective effects on bacterial composition and degradation potential. Diesel (regular fuel; cetane N^o^ 52; 9 PPM sulphur; obtained from an Esso fuel station) was used as it represented an extensive range of aliphatic and aromatic HCs. The ‘model oil’ composition has been described previously (Ferguson *et al.*^2017^) and is a simplified representative model of Schiehallion crude with a broad range of aliphatics (61%), aromatics—including monoaromatic (hereafter referred to as BTEX) and PAH (35%) and a resin fraction (4%). To assess HC degradation and changes in community structure, nine treatments were applied, including a crossed combination of HC mixture and consortia plus controls (Table S2, Supporting Information, lists all treatments). HCs were aseptically amended to 150 mL Erlenmeyer flasks containing 40 mL ONR7a medium and shaken at 150 RPM for 1 h to ensure dispersion and solubilisation within the medium. Frozen 500 µL enriched consortia glycerol stocks were resuscitated in 50 mL marine broth (hereafter referred to as MB, made up as per manufacturer instructions; Difco 2216, SLS Ltd, UK) for 30 h, to allow growth of slow- and fast-growing marine microbes, by incubation at 20°C on a rotary shaker at 150 RPM. The resuscitated consortium was then dispensed into ONR7a/HC flasks at a final concentration of 1% vol/vol and capped with a foam bung. Incubations were performed at 20 ± 2°C at 150 RPM for 42 days under dark conditions. Such a temperature was chosen, (1) to be different to the *in situ* temperatures to comparatively assess the effect of community composition on HC degradation potential and, (2) to represent a good approximate temperature for psychrophilic/tolerant organism growth. Therefore, degradation rates may be higher in this study than expected *in situ*. Triplicate samples were set up for destructive sampling at four time points (days 0, 7, 21 and 42). One mL samples were taken for enumeration of cultivable microbes (colony forming unit (CFU) counts) and bacterial isolation, basal respiration analysis, molecular analysis and the remainder was used for HC analysis.

### HC extraction and quantification

Residual HCs (both model oil and diesel) were extracted at all time points using 60 mL dichloromethane (DCM) in 6 × 10 mL sequential extractions. The 10 mL DCM were added to the flask and agitated for 30 min. Solvent/media mixture was then decanted into a separating funnel and shaken vigorously and left to separate for 10 min. Following separation, the organic phase was drained into a pre-weighed beaker. This was repeated 5 times, each time rinsing the incubation flask with solvent before adding it to the separating funnel, until collection of the final combined solvent phase and HC mixture. Pristane (>99%, Sigma Aldrich) was added prior to extraction as the surrogate standard to calculate extraction efficiency. Toluene (99.8%, Sigma Aldrich) was added as an internal standard at a final concentration of 0.1% vol/vol immediately prior to quantification. Loss of diesel was assessed gravimetrically. A 10 mL aliquot was taken and evaporated until constant dryness. Then degradation calculated as explained in Onur, Yilmaz and Icgen (2015). Degradation of model oil was quantified by separating the extract (1 µL) using gas chromatography (GC; Varian CP3800 fitted with 30 m Zebron ZB-50 column) and individual HCs were detected by a flame ionisation detector (FID). The conditions for the analysis were as follows: injector and detector temperature was 330°C using a split ratio of 5:1, initial oven temperature was 50°C with a 3 min hold and then increased at 10°C min^−1^ to 110°C, followed by and increase to 200°C at 5°C min^−1^ with at 12 min hold, finally the temperature was increased to 300°C at 20°C min^−1^ and held at 300°C for 6 min. HCs were quantified against an external calibration standard containing known amounts of the model oil components.

### Basal respiration analysis

To assess microbial respiration rates of the culture at specified time points, a 1 mL sample from each replicate was aliquoted into a vacuette (Greiner Bio-one, UK) and incubated at 20°C for 6 h. Following incubation, a 0.5 mL sample was taken from the headspace using an airtight Hamilton syringe and injected into a GC fitted with a methaniser and FID (Chrompack 9001). Analytical grade standards of 350, 1000, 3000, 5000 and 10 000PPM CO_2_ (Linde Group, UK) were used to generate a calibration curve to quantify injected samples.

### pH analysis

The pH of media was measured (Hanna Instruments pH209) at each sampling time point. The pH meter was calibrated against standards of pH 4, 7 and 10. Flasks were mixed whilst pH reading was taken to ensure the culture was homogenous.

### CFU counts and isolation of bacterial strains

To determine CFU counts and to isolate pure bacterial strains, 8-fold serial dilutions were performed in ¼ strength sterile Ringers solution (OXOID, UK). Aliquots from each dilution were spread onto marine agar (MB + 1.5% wt/vol bacteriological agar, OXOID, UK) for heterotrophic prokaryotes (HP) and ONR7 + 1% vol/vol corresponding HC source agar plates for hydrocarbon degrading prokaryotes (HDP). Single colonies were counted and CFU mL^−1^ media were estimated. Colonies with distinct morphologies were picked and subsequently purified by repetitive streaking onto marine agar and pure colonies were stored at −80°C in sterilised glycerol (∼17% final concentration).

### Taxonomic identification of bacterial isolates

Isolated bacterial strains were grown overnight in MB until the logarithmic growth phase had been reached. A 1.8 mL aliquot was taken for DNA extraction using UltraClean Microbial DNA isolation kits (Mo Bio, Carlsbad, CA, USA). The 16S rRNA gene was amplified by PCR using 27F and 1492R primers (Sigma Aldrich, UK) (Lane 1991). Reactions contained 1 µL of target DNA extract (∼20 ng µL^−1^), 22 µL Red Taq DNA Polymerase Master Mix with 1.5 mM MgCl_2_ (VWR, UK) and 1 µL of 10 µM of each primer and 1 µL of PCR H_2_O. Amplification was carried out on a Techne thermal cycler using the following programme: initial denaturation at 94°C for 5 min, followed by 25 cycles of 94°C for 60 s, 55°C for 60 s and 72°C for 60 s, plus final extension for 10 min at 72°C. PCR products were purified using Omega EZNA cycle purification kits (Omega Bio-Tek, Norcross, GA, USA) and sequenced by Sanger sequencing (Eurofins genomics, Germany). Sequences were quality checked using SeqTrace v 0.9.0 (Stucky 2012) and run through the Basic Local Alignment Search Tool (BLASTn) for nucleotide closest match. The ability of isolated bacterial strains to degrade HCs and reduce surface tension was assessed (see Supplementary Methods, Supporting Information).

### DNA extraction and Denaturing gradient gel electrophoresis

DNA was extracted from culture using the UltraClean Microbial DNA isolation kits (MoBio, Carlsbad, CA, USA) as per manufacturer instructions. DNA was quantified using a spectrophotometer (NanoDrop ND-1000). Nested PCR was performed adopting a first round of PCR using 27F and 1492R primers (Sigma, UK) (Lane 1991) to amplify full length 16S rRNA gene sequences as described above. The second round of PCR targeted the V3–5 region of the 16S rRNA gene using primer pair 341F (with GC clamp) and 907R (Sigma Aldrich, UK) (Muyzer and Smalla 1998) with the following programme: initial denaturation at 94°C for 4 min, followed by 35 cycles of 94°C for 30 s, 59°C for 30 s, and 72°C for 60 s, plus final extension for 10 min at 72°C. All PCR products where checked on a 1.2% agarose gel.

DGGE was performed using DCode Universal Mutation Detection System (Bio-Rad, Hertfordshire, UK) on the PCR amplified products for each station separately (i.e. one gel per station). Six µL of PCR product were loaded onto a 6% (wt/vol) polyacrylamide gel with a denaturant gradient of 30%–70% (100% corresponds to 7 M urea and 40% vol/vol formamide). Gels were electrophoresed in 1x TAE buffer at 60°C for 16 h at 75 V and silver stained, scanned and analysed with Phoretix 1D advanced analysis package (version 4.0; Phoretix Ltd., UK) as previously described (McCaig, Glover and Prosser 2001).

### Illumina sequencing of bacterial 16S rRNA genes

DNA extracts were PCR amplified using the KAPA Hi-Fidelity enzyme (Roche Diagnostics, UK) as previously described (Ferguson *et al.*^2017^). Pair-ended (2 × 300bp) amplicon sequencing across the V3–V4 region of the 16S rRNA gene was carried out on the Illumina MiSeq platform (Centre for Genome Enabled Biology and Medicine, University of Aberdeen). Average sample read depth was 43044 (± 2042 standard error; 88 samples). Bioinformatics analysis was carried out on the Maxwell high performance computing cluster at the University of Aberdeen, using Mothur v1.39.0 (Schloss *et al.*^2009^) with alignment and taxonomic assignment against the SILVA database (Quast *et al.*^2012^), with classification using the RDP Naive Bayesian classifier (Wang *et al.*^2007^), and with chimera removal using UCHIME (Edgar *et al.*^2011^). OTU clustering was performed at 97% similarity. The raw sequencing data is available in the European Nucleotide Archive under the accession number PRJEB26720.

### Statistical analysis

To assess differences in total petroleum hydrocarbons (TPH) degradation over time between the two stations and treatment, analysis of variance was tested using the formula: percentage of oil lost ∼ (Oil type + Treatment + Day + Station)^3^ (only three-way interactions were assessed as model optimisation deemed a four way interaction non-significant; Info 1, Supporting Information). To test for differences in individual HC degradation by day 42 between the two stations Welch’s t-test was performed with confidence intervals of 0.95. To compare basal respiration over time and between station andtreatment, analysis of variance was adopted with the following formula: }{}{\sqrt {\rm{Basal\,Respiration}}}\, \sim \, {\rm{Oil\,type}} \times {\rm Day} \times {\rm Station}. Respiration values were square-root transformed to meet model assumptions of homoscedasticity and normality (Info 2, Supporting Information).

Phoretix 1D advanced analysis package was used to produce relative band intensities within lanes of DGGE images. The statistical analysis of microbial communities from DGGE images was performed using the vegan package (Oksanen *et al.*^2013^) in R (R Core Team 2017). A distance matrix was generated for stations FSC135 and FSC1000 separately using the Bray–Curtis method from the relative band intensity data using the vegdist() function. Non-metric multi-dimensional scaling (nMDS) was adopted to visualise the effect of treatment on the microbial community structure using the metaMDS() function on the distance matrix. Hierarchical clustering was performed to identify similar groups using the hclust() function utilising the unweighted pair group method with arithmetic mean (UPGMA). To test for differences in microbial communities between treatment and over time, permutational multivariate analysis of variance (PerMANOVA) using relative band intensity distance matrices was performed using the adonis() function with 999 restarts.

Statistical analysis performed on Illumina sequencing data was carried out in R (R Core Team 2017). The finalised OTU table was imported into R using function import_biom() with the phyloseq package (McMurdie and Holmes 2013). Samples were not rarefied to retain maximum sequence data. Relative abundance plots were developed in phyloseq and visualised using the ggplot2 package (Wickham and Chang 2009). Alpha diversity measures were performed using plot_richness(). For beta diversity analysis a distance matrix was produced using function veg.dist() with the Bray–Curtis index and nMDS carried out using metaMDS() in vegan (Oksanen *et al.*^2013^). To correlate changes in microbial communities with HC removal, generalised additive models were fitted to nMDS plot using ordisurf() in vegan. To examine differences between community composition of samples based on treatment, station and time, PerMANOVA was performed on the distance matrix with 999 restarts.

## RESULTS

### Degradation of HCs by consortia

**Figure 1.**
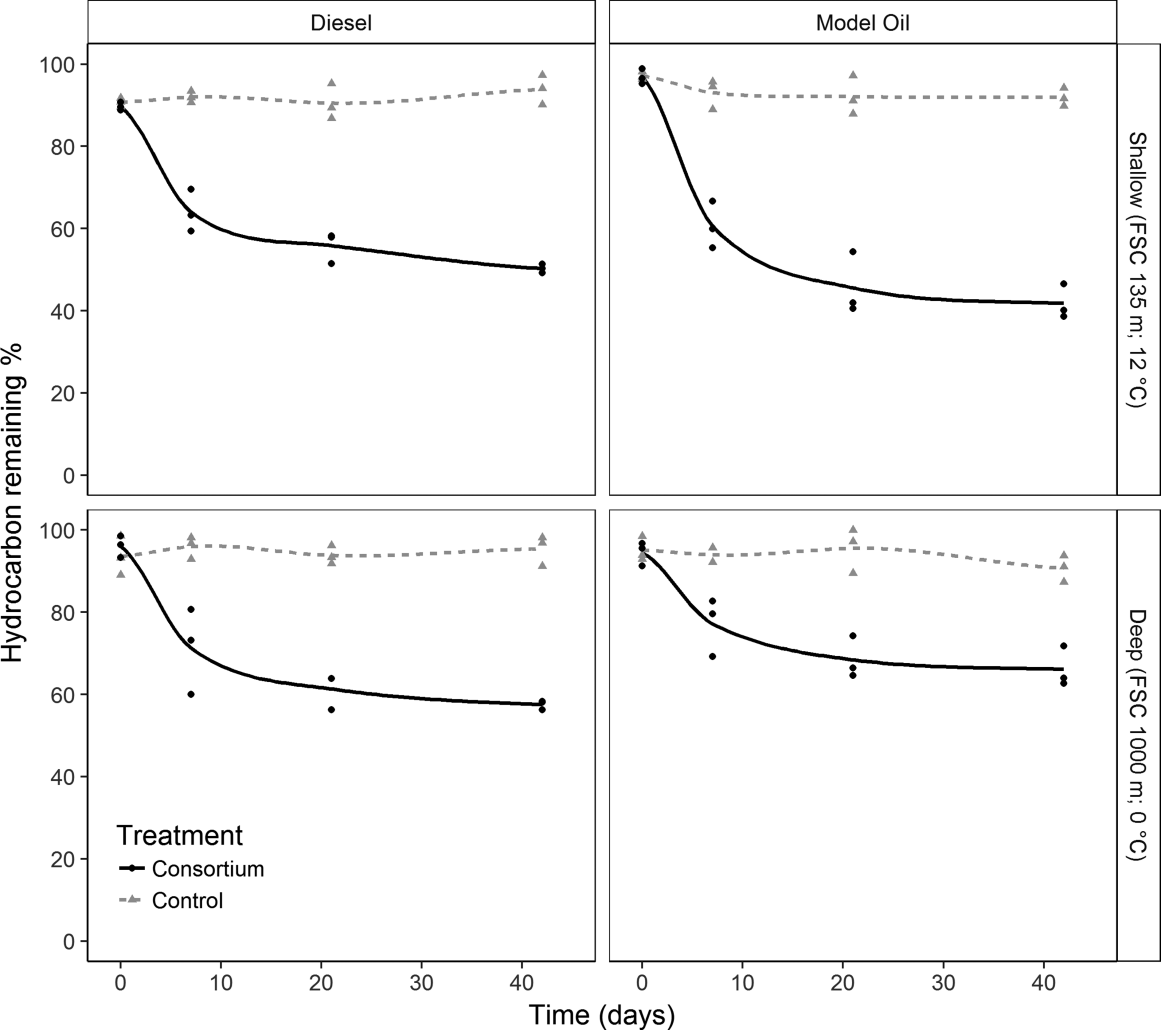
Degradation of TPH during incubations at 20 °C. Incubations were performed in triplicate. Triangles represent HC loss values of control treatments (no consortium added) and circles represent consortia inoculated treatments. Locally weighted regression (loess) smoothing was adopted to fit control (dashed line) and consortia (solid line) degradation profiles. Original station depth and temperature are included on the right y-axis.

Microbial consortia enriched from sediments at two stations within the FSC were incubated with two HC sources as the sole carbon source. Incubation of FSC135 and FSC1000 consortia with model oil resulted in significant HC removal by day 42 (both *P* < 0.001; Fig. 1, Info 1, Supporting Information). Model oil TPH was degraded significantly more by FSC135 (58.2 ± 2.4%) than FSC1000 (33.3 ± 2.9%; *P* = 0.003). Removal of aliphatic HCs was higher than that of PAHs in both stations (Table S3, Supporting Information). Aliphatic degradation rate decreased with increasing time and carbon chain-length (Table S3, Supporting Information; Fig. S1, Supporting Information). Degradation of diesel was significantly higher in FSC135 incubations compared to FSC1000 (*P* = 0.001) with removal of 49.7 ± 0.6 and 42.5 ± 0.6% respectively (Fig. 1). Most HC degradation in both stations occurred within 7 days of all treatments and throughout the experiment HC removal was significantly higher in biotic treatments compared to HC-only sterile (abiotic) treatments (Fig. 1; *P* < 0.001). BTEX compounds and the aliphatics, decane and 1-decene mostly volatilised within 7 days (determined by abiotic controls) and were excluded from further analysis.

Total aliphatics and PAHs in model oil treatments were degraded significantly more by FSC135 than FSC1000 (*P* = 0.003 and *P* = 0.004 respectively); however, several higher molecular weight HCs such as pyrene were degraded to similar levels (Table S3, Supporting Information). For FSC135, the shortest chained aliphatic HC dodecane, was the most degraded (85.8 ± 2.1%) while the longest chained aliphatic, tetracosane was the least degraded (40.1 ± 5.3%; Table S3, Supporting Information and Fig. S1, Supporting Information). Within PAHs, naphthalenewas almost entirely degraded (99.2 ± 0.8%) whereas pyrene showed the least degradation (29.6 ± 7.3%) after 42 days. In contrast, in FSC1000, phenanthrene was the least degraded PAH (13 ± 5%) in FSC1000.

### Microbial consortia activity

Basal respiration was monitored to assess production of CO_2_ due to mineralisation of HCs as the sole source of carbon. Respiration values significantly changed over time (*P* < 0.001) with highest values on day 7 for model oil treatments at 1.17 ± 0.12 and 1.17 ± 0.14 (error = SE; *n* = 3) mg CO_2_ mL^−1^ media day^−1^ for FSC135 and FSC1000 stations, respectively. Values then rapidly decreased by day 42 where levels were similar to those of day 0 (Fig. S2, Supporting Information). A similar pattern was observed in diesel incubations where respiration values were highest at day 7 at decreased by day 42.

Microbial enumeration was estimated by CFU counts at all time points. Counts for HP were consistently higher than for HDP in all treatments and stations. Abundance of HP ranged from 10^8^ to 10^10^ CFU mL^−1^ and from 10^6^ to 10^8^ CFU mL^−1^ for HDP (Table S4, Supporting Information). For FSC135 and FSC1000, counts of HP were highest at day 7 in both HC treatments (except: FSC1000 diesel = day 0). For HDP, highest counts were recorded at day 21 for both HC treatments (except: FSC1000 diesel = day 7). There was no growth in any of the control treatments.

Measurements of culture medium pH were taken to monitor evolving conditions. The initial pH of the mineral seawater medium ONR7a was adjusted to 7.4 as according to Dyksterhouse *et al.* (1995). The pH of the control cultures remained relatively constant at all time points within a range of 7.2–7.6. In treatments with FSC135 and both HC sources, the pH decreased to ∼5.5 by day 7 and remained stable. The pH recorded in FSC1000 and HC sources reduced less rapidly to ∼6.5 by day 7 and then to ∼5.5 by day 21 and remained stable.

### Analysis of bacterial communities

Variation of microbial community structure over time (assessed by DGGE) demonstrated clustering of communities by treatment and time in both stations (Figures S3 and S4, Supporting Information). PerMANOVA analysis showed a significant difference in community composition based on the interaction of treatment and time, in both FSC135 (*P* = 0.003; Info 3, Supporting Information) and FSC1000 stations (*P* < 0.001; Info 4, Supporting Information).

**Figure 2.**
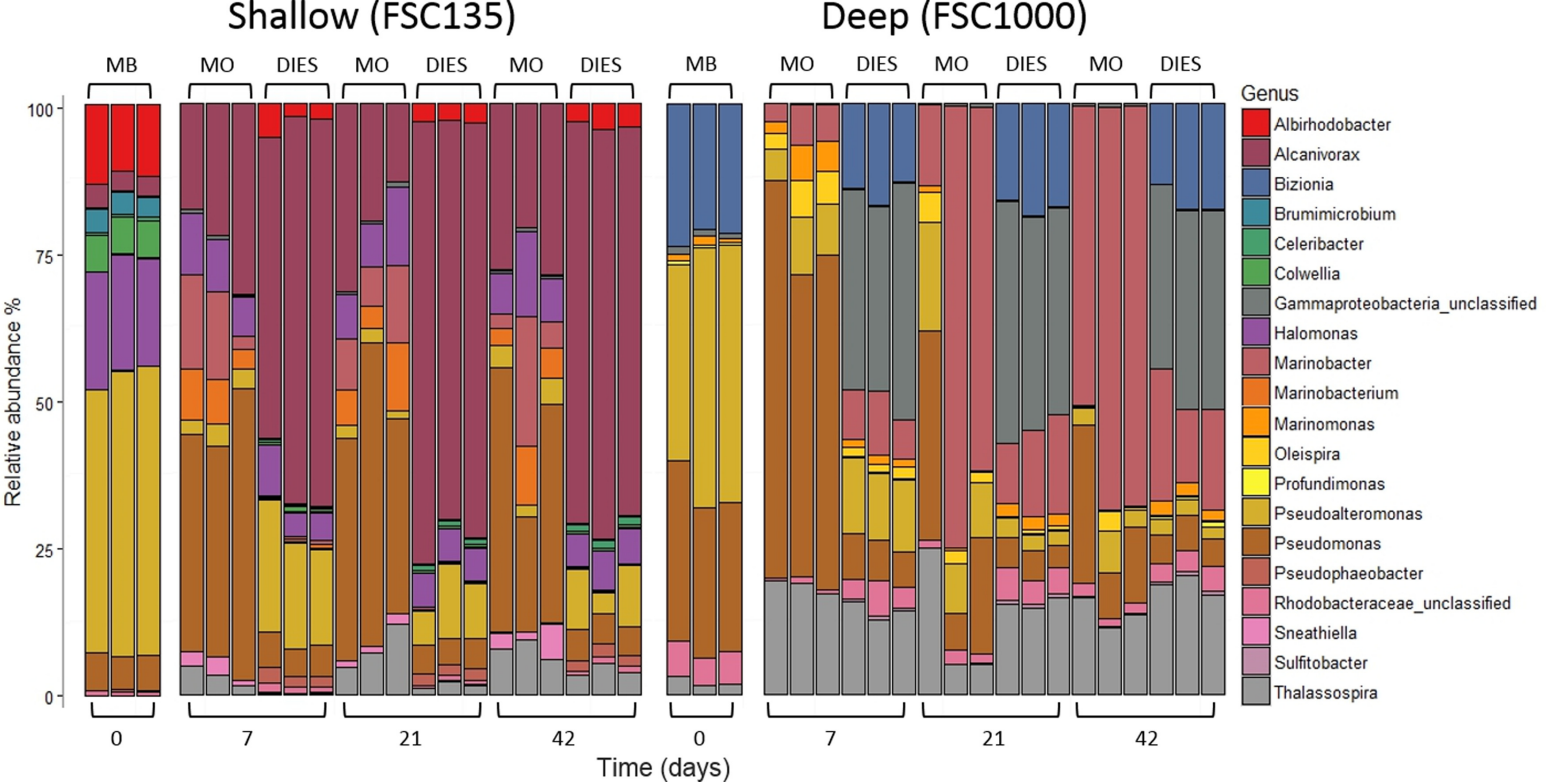
Relative abundance plots visualising bacterial community composition and temporal dynamics. Bars represent top 20 OTUs for each sample grouped at the genus level in triplicate. On the upper x-axis, MB indicates marine broth resuscitated community, MO and DIES represents model oil and diesel treated communities respectively.

Microbial community composition of consortia was analysed by Illumina sequencing of the V3–4 region of the 16S rRNA gene. Controls without HC were not analysed as there was no indication of growth. The HC-degrading consortia enriched from both stations were dominated by Proteobacteria (95% in FSC135; 77% in FSC1000). *γ*-Proteobacteria, particularly *Pseudoalteromonas* (45%) and *Halomonas* (18%), dominated the FSC135 consortium (Fig. 2). Less prevalent genera included *Colwellia* (6%), *Pseudomonas* (6%) and *Alcanivorax* (3%). The FSC1000 consortium differed in composition where *Pseudoalteromonas* and *Pseudomonas*equally dominated the community (both 30%). From *α*-Proteobacteria, *Albirhodobacter* (13%) and *Bizionia* (22%) were present in FSC135 and FSC1000 communities respectively.

**Figure 3.**
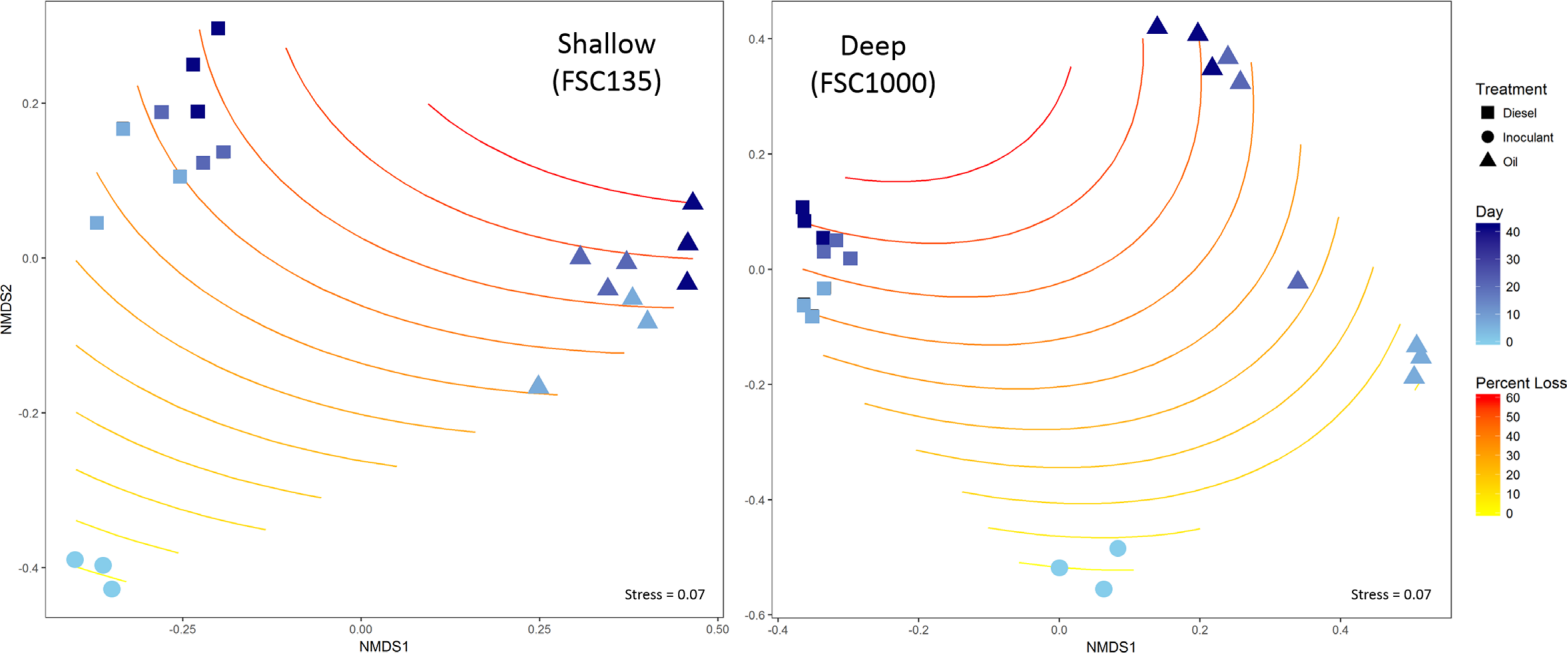
Ordination (nMDS) plots based on the Bray–Curtis dissimilarity index of bacterial communities in shallow (FSC135; left) and deep (FSC1000; right) stations. Darker shades (blue) of plot points indicate an increase in time (days) as indicated by the ‘Day’ gradient scale. Isolines indicate percentage of HC degraded over time using generalised additive models with darker-shaded lines (red) representing higher percentages of HC loss as indicated by the ‘Percent Loss’ gradient colour scale.

As demonstrated by DGGE, analysis of the Illumina sequencing results revealed that community composition changed significantly following incubation with both HC treatments, in both stations, over time (*P* = 0.009). Ordination analysis (nMDS) revealed dissimilarity of communities between station and treatment (Fig. S5, Supporting Information). To visualise the relationship between changes in community structure of each station over time with removal of HCs, an nMDS was constructed and fitted with isolines (generalised additive models representing percentage loss of HCs) that indicated decreasing HC levels (Fig. 3). Changes in community composition over time corresponded with increasing HC removal rates.

Overall there were distinct differences in the community composition of the two stations and noticeable variation between the two HC mixtures (Fig. 2 and Fig. S6, Supporting Information). Incubation of the FSC135 consortium with model oil resulted in a decrease in the relative abundance of *Pseudoalteromonas* and *Albirhodobacter*, whereas *Pseudomonas* and *Alcanivorax* (40% and 23% respectively at day 7) were selected for and dominated the community (Fig. 2). This contrasted with diesel-treated communities where *Alcanivorax* (60%) dominated the community at day 7 and *Pseudomonas* (5%) was less prominent. *Halomonas* (∼7%) was the only genera that was similarly affected by both treatments and remained present throughout the study.

Station FSC1000 demonstrated disparities in community structure depending on HC treatment (Fig. 2 and Fig. S6, Supporting Information). *Pseudoalteromonas* gradually decreased in relative abundance in both treatments. *Pseudomonas* was initially enriched in model oil treatments (up to 60% at day 7) but was then succeeded by *Marinobacter* (60% at day 42). *Thalassospira*, an *α*-Proteobacterium, was enriched in both treatments (up to 25%) but exhibited no trend over time or treatment. *Oleispira* was enriched by model oil also (5% at day 7). Diesel-treated communities were made up of several taxa including *Bizionia*, *Thalassospira* and *Pseudomonas*, which were present at all time points albeit at lower relative abundance compared to model oil. A large proportion of the genera present in diesel treated communities were unclassified *γ*-Proteobacteria (34% at day 7).

### Isolation of pure bacterial cultures from consortia

To study HC degraders from the FSC in more detail, 34 and 36 morphologically different strains were isolated from consortia FSC135 and FSC1000, respectively. All FSC135 strains were affiliated to Proteobacteria, among which 32 were *γ*-Proteobacteria (8 *Pseudoalteromonas*, 11 *Halomonas*, 4 *Pseudomonas* and 3 *Alcanivorax)* and 2 *α*-Proteobacteria (*Pseudorhodobacter incheonensis* strain KOPRI (FSC135.16; nucleotide identity 99%) and *Thalassospira alkalitolerans* strain MBE#61 (FSC135.49; 99%)) (Table S5, Supporting Information).

FSC1000 strains comprised Bacteroidetes (2) and Proteobacteria (32). Isolates affiliated with *γ*-Proteobacteria (28) include *Marinobacter* (13), *Pseudoalteromonas* (10), *Pseudomonas* (3) and *Marinomonas* (1). Four strains matched to *α*-Proteobacteria and were identified as *Thalassospira* (3) and *Sulfitobacter* (1). Two strains were affiliated with *Bizionia* (both 99%) of Bacteroidetes (Table S6, Supporting Information). Strains isolated from FSC1000 consortium were tested for their ability to utilise diesel as a sole carbon source and to reduce surface tension (see Supplementary Results, Supporting Information).

## DISCUSSION

Bacterial oil degradation is regulated by a plethora of environmental factors (Duran and Cravo-Laureau 2016; Liu, Bacosa and Liu 2017). In this study, the effect of sediment bacterial community composition on maximal HC degradation potential was evaluated. Microbial consortia from two sediment depths (135 m and 1000 m water depth) of the FSC were enriched with crude oil at *in situ* temperatures (0°C and 12°C for the deep and shallow stations, respectively); this supported acclimatisation of indigenous microbial communities to a diverse range of HCs. Consortia enriched from shallow sediments exhibited greater HC (model oil and diesel) maximal degradation potential than deep sediment consortia over 42 days, despite incubation at an equivalent temperature (20°C). Considering the incubation temperature, the degradation potential estimated in this study should be interpreted cautiously as lower rates would be expected under *in situ* conditions.

### Effect of geographical origin on HC degradation potential

Higher HC degradation by the shallow station (FSC135) consortium compared to the deep (FSC1000) station (at 20°C) indicates that differences in site-specific community structure can influence maximal biodegradation potential. Deep-sea microbial communities often exhibit lower HC degradation potential than shallower communities when incubations are performed at *in situ* temperatures (Campo, Venosa and Suidan 2013; Ferguson *et al.*^2017^; Techtmann *et al.*^2017^); this suggests that HC degradation is linked to temperature with lower degradation potential being common with lower temperatures (Coulon *et al.*^2007^; Bargiela *et al.*^2015^). However, degradation ability is also probably dependent on microbial community composition, resulting from long-term adaptation to a specific environment. In particular, *in situ* temperatures provide a specific environmental niche that drives aspects of community selection and microbial specialisation, in turn explaining different degradation rates (Coulon *et al.*^2007^; Redmond and Valentine 2012). HC degradation of the deep community (FSC1000) in this study was higher than that observed at *in situ* temperatures (Ferguson *et al.*^2017^), supporting the argument that higher temperatures (20°C instead of 0°C) increase degradation rates due to higher microbial growth (and associated metabolic rates).

A microbial community can be influenced by numerous environmental factors that, in turn, influences HC degradation potential. For example, microbial communities from chronically polluted sites may respond faster to HC exposure (Bargiela *et al.*^2015^). Overall TPH degradation observed in this study (61% in FSC135) was lower than the rate demonstrated by a consortium enriched from sediments exposed to HCs following the *Prestige* oil tanker incident (Northern Spain, 2002) (81% degradation) (Vila *et al.*^2010^). Indeed, FSC sediments are not known to be contaminated with HCs (Perez Calderon *et al.*^2018^) and may not be primed to degrade HCs. Additionally, environmental conditions present *in situ* can hinder degradation potential. Seabed anoxia can develop following oil residue and marine snow deposition due to benthic smothering and oxygen consumption (Hastings *et al.*^2016^). This delays HC removal as anaerobic degradation is typically slower than aerobic (Widdel, Knittel and Galushko 2010). Increased hydrostatic pressure is also known to impact microbial functioning and specific adaptations are required to withstand pressure-induced stress (Oger and Jebbar 2010). It has been demonstrated that increasing hydrostatic pressure can decrease HC-degrading bacterial growth rates and degradation capacity (Schedler *et al.*^2014^; Marietou *et al.*^2018^). Additionally, organic matter availability and type can vary with regard to increasing marine depth (Orcutt *et al.*^2011^), which may also be influenced by marine snow deposition and will likely alter microbial functioning.

### Influence of HC substrate on degradation potential

Degradation of HCs typically follows a sequential pattern of low to high molecular weights as previously demonstrated (Brakstad, Nordtug and Throne-Holst 2015; Bagby *et al.*^2017^). Here, although lower molecular weight HCs were degraded more than higher molecular weight HCs, degradation of all HCs appeared to occur simultaneously over time (Fig. S1, Supporting Information). HC structural complexity could explain slower degradation rates of heavier molecular weight HCs, which, may appear as sequential degradation from lower to heavier weight HCs (Head, Jones and Röling 2006). Shorter-chained aliphatics are usually more bioavailable than long chain aliphatics due to higher water-solubility; therefore, they are among the HCs to be degraded most rapidly (Rojo 2009; Techtmann *et al.*^2017^). In this study, shorter-chained aliphatics including dodecane and tetradecane underwent the highest levels of degradation, whereas long chained docosane was less degraded. These patterns are consistent with findings *in situ* (Bagby *et al.*^2017^).

In both consortia, degradation of aliphatics was higher compared to aromatics in model oil treatments; however, naphthalene underwent near complete degradation, which is probably a consequence of its relatively high water-solubility, making it more accessible to microbes in culture. Indeed, naphthalene is readily degraded in both arctic and temperate environments (Bagi *et al.*^2014^). Degradation of aromatic HCs in this study was relatively high compared to previous studies, probably due to a lower proportion of recalcitrant PAHs (Díez *et al.*^2005^; Vila *et al.*^2010^). The extent and rate of aromatic HC degradation commonly decreases with increasing ring number. Pyrene is the largest PAH structurally in the model oil (4 benzene rings) and was least degraded within the FSC135 station. However, in the FSC1000 station, dibenzothiophene was least degraded (20%); in contrast to FSC135 station where it was degraded by 44%. Dibenzothiophene is considered relatively degradable by organisms that harbour specific desulfurisation metabolic pathways (Duarte *et al.*^2001^; Khedkar and Shanker 2014) and results in production of metabolites such as 3-Hydroxy-2-formylbenzothiophene (Mormile and Atlas 1988). The presence of these metabolites can cause culture medium to turn reddish-purple (Bressler and Fedorak 2001; Khedkar and Shanker 2014). This was observed in FSC135 cultures (Fig. S7, Supporting Information), indicating enhanced breakdown of dibenzothiophene; however this was not observed in FSC1000 cultures and probably implies lower degradation of dibenzothiophene.

### Microbial community activity associated with HC degradation

HC degradation is typically assumed to follow first order kinetics, where the majority of removal occurs early in the process (Thessen and North 2016; Wang *et al.*^2016^). Accordingly, degradation in this study was most prominent within 7 days (Fig. 1). Estimated microbial activity and abundance also identified day 7 as the most active stage of the incubation period. Rates in microbial activity and abundance were consistent with changes in pH, itself potentially due to production of acidic aromatic metabolites (Peng *et al.*^2008^) or acidic surfactants (Paulino *et al.*^2016^). This pH reduction may have had limiting effects on microbial functioning and subsequently degradation ability as biodegradation is known to be optimal at a pH range of 6–9 (Das and Chandran 2011). These temporal patterns were also seen in bacterial community composition whereby there was a constrained amount of change following the first week of incubation (Fig. 2).

### Influence of geographical origin and HC substrate on bacterial community structure

The incubation of two microbial consortia with two HC mixtures induced selection of distinct bacterial communities. This supports previous evidence that community composition can be dramatically altered when exposed to different HC structures (McKew *et al.*^2007^; Viggor *et al.*^2013^). Within the shallow station consortium the relative abundance of *Alcanivorax* increased rapidly following incubation with diesel. Commonly, *Alcanivorax* are present in low abundance in non-contaminated environments, only to bloom and dominate microbial communities when exposed to HCs (Harayama and Kishira 1999; Hara, Syutsubo and Harayama 2003). Certain *Alcanivorax* strains are more suited to temperate (Gertler *et al.*^2012^) and atmospheric pressure (0.1 MPa) environments (Scoma *et al.*2016a,b); which probably explains why *Alcanivorax* was not detected in the consortia issued from deep station. The lack of hydrocarbonoclastic bacteria in the FSC1000 consortium exposed with HC is consistent with findings from *in situ* studies following DWH (reviewed in Scoma, Yakimov and Boon 2016). Therefore, high maximal degradation potential by the shallow consortium may be explained by the dominance of *Alcanivorax* spp, as they are proficient alkane degraders (Harayama, Kasai and Hara 2004; Yakimov, Timmis and Golyshin 2007; Kostka *et al.*^2011^).

Model oil strongly selected for *Pseudomonas* in both the FSC135 and FSC1000 communities. Stronger presence of *Pseudomonas* in model oil treatments compared to diesel treatments could be explained by *Pseudomonas*’ ability to degrade BTEX compounds (Giudice *et al.*^2010^), which were present in the model oil. Its presence in both stations and treatments is testament to its ubiquity and versatility and could be crucial in oil spill remediation. *Marinobacter* appeared to succeed *Pseudomonas* in the model oil-treated FSC1000 community, indicated by the high variation between replicates at day 21. Interestingly, *Marinobacter* was detected in the late successional phase of biodegradation experiments using Macondo oil (Brakstad *et al.*^2015^), and was hypothesised to coincide with degradation of PAHs or longer-chained aliphatic HCs, once lower molecular weight HCs were consumed.

Certain taxa were present in only one consortium. *Halomonas* was detected in FSC135 communities throughout the incubation period but decreased in relative abundance following HC amendment. This is surprising considering *Halomonas* is frequently implicated in HC degradation (Mnif, Chamkha and Sayadi 2009) and flourishes in oil-contaminated environments (Hazen *et al.*^2010^). HC-degrading *Oleispira* spp. are often detected in cold marine environments (Yakimov, Timmis and Golyshin 2007) and was present in model oil amended deep station communities. Microbial communities from DWH water column samples (1070 m deep) were dominated by *Oleispira*-related OTUs when incubated at 3 different hydrostatic pressure levels (0.1, 15 and 30 MPa) at 4°C with Macondo crude oil (Marietou *et al.*^2018^). Furthermore, single cells obtained from the DWH oil plume were matched (97%) to *Oleispira antarctica* (Mason *et al.*^2012^) that suggests a role for *Oleispira*-related strains in a deep-sea response. *Colwellia* was present at day 0 in the FSC135 consortium but decreased in relative abundance over time and may have been outcompeted by taxa suited to higher temperatures considering its adaptation to cold environments (Methé *et al.*^2005^). It is therefore surprising that *Colwellia* was not enriched in the FSC1000 station, particularly as it strongly dominated oil-contaminated deep FSC sediments obtained from the same location incubated at 0 °C (Perez Calderon *et al.*^2018^) and was enriched in DWH plume samples (Redmond and Valentine 2012).

Members of *α*-Proteobacteria were present in both stations and are commonly linked to aromatic HC degradation (Alonso-Gutiérrez *et al.*^2009^; Gertler *et al.*^2012^). *Thalassospira* increased in relative abundance following HC treatment in both stations indicating HC utilisation and has previously demonstrated phenanthrene (Gutierrez *et al.*^2015^) and pyrene (Zhou *et al.*^2016^) degradation. In preliminary biosurfactant production tests, *Thalassospira* was the most promising candidate (see Supplementary Results, Supporting Information), although further work must be performed to sufficiently test production ability. Producing surfactants aids in the uptake of HCs by the producing organism and the community as a whole (Ron and Rosenberg 2002; Gutierrez *et al.*^2013^) and is an important parameter to consider in biodegradation studies. *Bizionia* was selected in diesel-treated FSC1000 communities and are commonly found in cold marine waters (Li *et al.*^2015^) and sediments (Zhang *et al.*^2017^). This genus is rarely described in oil degradation or contamination studies; however, *Bizionia* increased in relative abundance when sub-Antarctic sediment was exposed to crude oil (Guibert *et al.*^2012^). To our knowledge the cultured isolate (FSC1000.24; Table S6, Supporting Information) from this study represents the first *Bizionia* strain to be obtained from HC exposed samples. Furthermore, isolate FSC1000.24 was capable of growth on diesel as the sole carbon source (based on CFU count data, data not shown) when incubated individually. However, its growth was limited and further work is required to evaluate optimum growth conditions.

## CONCLUSIONS

Considering increased HC exploration in deep-sea environments, there is a need to further understand the behaviour and response of HC-degrading populations following oil contamination events. This study demonstrated that despite identical incubation conditions, a community from a deep-sea region was not as capable of HC degradation as a shallower region. This was concomitant with variations in community composition and although there were shared phylotypes between the two stations, there were certain site-specific taxa such as piezosensitive *Alcanivorax* that may have been responsible for increased HC removal in the shallower station. Thus, it was identified that microbial community composition, as well as other environmental parameters, such as oil type, may influence biodegradation potential. From this, it was inferred that site-specific responses to oil contamination would lead to variations in HC degradation potential, particularly at *in situ* conditions when temperature and hydrostatic pressure are influencing factors. Further work using techniques to more directly link microbial community structure to HC degradation at *in situ* conditions such as stable isotope probing approaches will develop our understanding of these processes.

## Supplementary Material

Supplementary Data
