# Supplementary material for: Effect of spatial origin and hydrocarbon composition on bacterial consortia community structure and hydrocarbon biodegradation rates
Source: FEMS Microbiol Ecol. 2018 Jun 30;94(9):fiy127. doi: 10.1093/femsec/fiy127 (PMC6166136; doi:10.1093/femsec/fiy127)
Supplement: Supplementary Data [file fiy127_supplemental_files.zip › Supplementary_Potts.docx]

**SUPPLEMENTARY MATERIAL**

**Supplementary Methods**

## *Assessment of FSC1000 bacterial isolates to utilise hydrocarbons and reduce surface tension*

Isolates were resuscitated from glycerol stocks on marine agar plates and a loopful of growth material inoculated into 10 ml MB and ONR7a + diesel and agitated at 200 RPM at 20 °C until turbid. To estimate growth rates, 1% vol/vol inoculum was dispensed into 250 ml flasks containing 100 ml MB in triplicate at 20 °C and 200 RPM. Optical density at 600 nm was measured every 2–4 hours depending on speed of bacterial growth using 1 ml of culture in spectrophotometer (Camspec M107) until stationary phase growth was evident. Growth rates (see Supplementary Table 7) were calculated as stated in Schlegel and Zaborosch (1993) using the equation:

$$\mu= \frac{{\log x}_{t}-{logx}_{0}}{loge(t-t_{0})} = \frac{{lnx}_{t}- {lnx}_{0}}{(t-t_{0})}$$

Where $x$ is bacterial density (optical density at 600 nm) and $t$ is time (hours). Subsequently, the ability of isolates to utilise diesel as a sole source of carbon was determined. Flasks containing ONR7a were ammended with 1% vol/vol filter-sterilised (PTFE 0.2 µl) diesel and inoculated with culture as before. Samples were taken for OD measurements as previously described. Furthermore, 1 ml was aliquoted for surface tension measurements using a tensiometer (White’s instruments) fitted with a Du Nouy ring. ONR7 and 1% v/v diesel without culture inoculum was used as a negative control. CFU counts were performed as described above to enumerate cultures. A wild type strain of *Alcanivorax borkumensis* SK2 (purchased from DSMZ) was used as a positive control for both diesel growth and surfactant production.

**Supplementary Results**

*Assessment of isolated FSC1000 strains for utilisation of hydrocarbons and reduction of surface tension*

Phylogenetically diverse bacterial strains isolated from FSC1000 were tested for hydrocarbon utilisation and biosurfactant production. All isolates showed ability to utilise diesel as the sole source of carbon, assessed by changes in turbidity of culture compared to a diesel only negative control. Although strain FSC1000.46 (*Thalassospira* sp. EM202) exhibited limited growth in MB, cultures became very turbid when incubated in ONR7a medium with diesel as sole substrate (data not shown). There was no significant reduction (p>0.05) in surface tension of seawater in any isolate compared to control samples without inoculant; however, strain FSC1000.46 did reduce surface tension by 6% (p = 0.061). Positive control *Alcanivorax borkumensis* SK2 significantly reduced surface tension by 26% (p<0.05), confirming suitability of the test.

Supplementary Table 1. Properties of samples obtained for enrichment. Samples were stored at 4 °C for transport to the laboratory (no longer than 2 weeks). Sediments were kept submerged in seawater and aerated.

| **Station** | **Depth**  **(m)** | **Longitude** | **Latitude** | **Temp (°C)** | **Salinity PSU** | **Dissolved oxygen mmol/L** | **Date** |
| --- | --- | --- | --- | --- | --- | --- | --- |
| FSC135 | 135 | 59°53.16’N | 03°20.52’W | 12.0 | N/A | N/A | Nov ‘13 |
| FSC1000 | 994 | 61°35.00'N | 04°15.00'W | -0.7 | 34.9 | 194.7 | April ‘14 |

Supplementary Table 2. Outline of treatments setup in triplicate.

|  | **No hydrocarbon** | **Diesel** | **Model oil** |
| --- | --- | --- | --- |
| No consortium | × | × | × |
| FSC135 consortium | × | × | × |
| FSC1000 consortium | × | × | × |

Supplementary Table 3. Comparison of overall hydrocarbon degradation between FSC135 and FSC1000 consortia. Significance tested by Welch two sample t-test, not significant: p >0.05, at 95 % confidence interval.

| **Oil type** | **Hydrocarbon** | **C*_n_*/**  **ring N^o^** | **FSC135**  **% loss ± SE (42 days)** | **FSC1000**  **% loss ± SE (42 days)** | **Difference p-value** |  |
| --- | --- | --- | --- | --- | --- | --- |
| Model oil | ∑TPH | - | 58.2 ± 2.4 | 33.3 ± 2.9 | 0.0031 | ** |
| Aliphatics | ∑Aliphatics | - | 61.1 ± 2.4 | 35.8 ± 2.7 | 0.0028 | ** |
|  | Dodecane | 12 | 85.8 ± 2.1 | 59 ± 3.4 | 0.0047 | ** |
|  | Tetradecane | 14 | 60.4 ± 2.5 | 33.6 ± 3.2 | 0.0032 | ** |
|  | Pentadecane | 15 | 54.8 ± 2.4 | 27.5 ± 3.6 | 0.0051 | ** |
|  | Hexadecane | 16 | 52.6 ± 2.4 | 24 ± 3.6 | 0.0046 | ** |
|  | Heptadecane | 17 | 54.4 ± 1.8 | 27.6 ± 3.8 | 0.0095 | * |
|  | 1-Octadecene | 18 | 49.9 ± 2.5 | 36.3 ± 4.7 | 0.0821 | ∙ |
|  | Eicosane | 20 | 42.8 ± 5.7 | 26.5 ± 3.7 | 0.0840 | ∙ |
|  | Docosane | 22 | 40.6 ± 4.7 | 25.4 ± 2.7 | 0.0623 | ∙ |
|  | Tetracosane | 24 | 40.1 ± 5.3 | 26.2 ± 1.6 | 0.1100 |  |
| Aromatics | ∑PAH | - | 54 ± 2.6 | 29.5 ± 3.5 | 0.0044 | ** |
|  | Napththalene | 2 | 99.2 ± 0.8 | 87.9 ± 1.5 | 0.0060 | ** |
|  | Fluorene | 3 | 47.8 ± 4.2 | 20.3 ± 4.0 | 0.0092 | ** |
|  | Phenanthrene | 3 | 44.3 ± 4.7 | 13 ± 5.0 | 0.0102 | * |
|  | Anthracene | 3 | 42.7 ± 5.6 | 49.4 ± 5.2 | 0.4267 |  |
|  | Dibenzothiophene | 3 | 44.5 ± 3.6 | 12.3 ± 2.3 | 0.0029 | ** |
|  | Fluoranthene | 4 | 39.5 ± 5.2 | 16.7 ± 2.4 | 0.0328 | * |
|  | Pyrene | 4 | 29.9 ± 7.3 | 19.3 ± 1.9 | 0.7427 |  |
| Diesel | ∑TPH | - | 49.7 ± 0.6 | 42.5 ± 0.6 | 0.0012 | ** |

Differences with p-values <0.1, <0.05, <0.01 and <0.001 are indicated with “∙”, “*”, “**” and “***”, respectively.

Supplementary Table 4. CFU counts. Red and green lettering indicates decrease and

increase, respectively, compared to previous time point value. Blue indicates time 0 value.

HP = heterotrophic prokaryotes

HDP = hydrocarbon degrading prokaryotes

|  |  | **Diesel** | | **Model oil** | |
| --- | --- | --- | --- | --- | --- |
| **Station** | **Days** | **HP** | **HDP** | **HP** | **HDP** |
| FSC135 | 0 | 3.44E+09 | 3.67E+06 | 3.44E+09 | 2.24E+06 |
|  | 7 | 1.22E+10 | 1.43E+07 | 1.64E+10 | 1.40E+07 |
|  | 21 | 1.10E+09 | 2.45E+07 | 5.00E+08 | 1.70E+07 |
|  | 42 | 1.33E+08 | 2.07E+07 | 4.87E+08 | 4.80E+06 |
| FSC1000 | 0 | 6.20E+09 | 4.85E+06 | 6.20E+08 | 1.32E+06 |
|  | 7 | 2.53E+09 | 1.46E+08 | 2.50E+09 | 6.40E+07 |
|  | 21 | 4.67E+08 | 6.30E+07 | 2.00E+09 | 8.50E+07 |
|  | 42 | 1.33E+08 | 4.93E+07 | 8.67E+08 | 2.50E+07 |

Supplementary Table 5. Identification of FSC135 station isolates using BLASTn

| **Isolate ID** | **Growth substrate** | **Day sampled** | **Closest relative (BLASTn** | **Similarity (%)** | **Accession N^o^** |
| --- | --- | --- | --- | --- | --- |
| 1 | MB | 0 | *Pseudoalteromonas issachenkonii strain R03-1* | 97 | JX170238.1 |
| 2 | MB | 0 | *Pseudoalteromonas sp. strain 8-16* | 99 | KX806624.1 |
| 3 | MB | 0 | *Pseudoalteromonas undina isolate M1* | 98 | LK391517.1 |
| 6 | MB | 0 | *Pseudoalteromonas sp. 114Z-11* | 99 | GU584139.1 |
| 7 | MB | 0 | *Alcanivorax borkumensis strain SK2* | 99 | NR_074890.1 |
| 9 | MO | 7 | *Halomonas glaciei strain DD 39* | 99 | NR_114866.1 |
| 10 | MO | 7 | *Halomonas meridiana strain X36* | 99 | JX122625.1 |
| 11A | MO | 7 | *Pseudoalteromonas sp. 66_9* | 98 | KP120841.1 |
| 11B | MO | 7 | *Halomonas neptunia strain MAT-17* | 99 | KC354707.1 |
| 12 | DIES | 7 | *Pseudoalteromonas elyakovii strain BSi20610* | 98 | DQ537520.1 |
| 14 | DIES | 7 | *Pseudoalteromonas undina strain XH124* | 99 | KC178900.1 |
| 15 | DIES | 7 | *Halomonas alkaliphila strain NJES-52* | 99 | KR140242.1 |
| 16 | DIES | 7 | *Pseudorhodobacter incheonensis strain KOPRI* | 99 | DQ001322.1 |
| 18B | DIES | 7 | *Halomonas neptunia strain MAT-17* | 99 | KC354707.1 |
| 18C | DIES | 7 | *Halomonas campaniensis strain LS21* | 99 | CP007757.1 |
| 21 | MO | 21 | *Pseudomonas sp. ice-oil-215* | 99 | DQ533960.1 |
| 22 | MO | 21 | *Halomonas glaciei strain DD 39* | 99 | NR_114866.1 |
| 23A | MO | 21 | *Salinibacterium amurskyense strain KMM 6705* | 99 | KC247355.1 |
| 24 | MO | 21 | *Pseudomonas pelagia strain TB-149* | 99 | KF993339.1 |
| 26 | MO | 21 | *Halomonas alkaliphila strain 18bAG* | 99 | NR_042256.1 |
| 29 | MO | 21 | *Pseudomonas sp. ESC-str.770* | 99 | HE586890.1 |
| 31 | MO | 21 | *Halomonas neptunia strain MAT-17* | 99 | KC354707.1 |
| 34 | DIES | 21 | *Halomonas neptunia strain MAT-17* | 99 | KC354707.2 |
| 38 | MO | 21 | *Pseudomonas sp. ESC-str.770* | 99 | HE586890.1 |
| 42 | MO | 42 | *Pseudoalteromonas tetraodonis strain VSG722* | 99 | KC534357.1 |
| 43 | MO | 42 | *Halomonas neptunia strain MAT-17* | 99 | KC354707.2 |
| 46 | MO | 42 | *Alcanivorax sp. P75* | 99 | EU195941.1 |
| 48 | MO | 42 | *Alcanivorax sp. NBRC 101098* | 99 | AP014613.1 |
| 49 | DIES | 42 | *Thalassospira alkalitolerans strain MBE#61* | 99 | NR_114386.1 |

Supplementary Table 6. Identification of FSC1000 station isolates using BLASTn

| **Isolate ID** | **Growth Substrate** | **Day sampled** | **Closest relative (BLASTn)** | **Similarity (%)** | **Accession N^o^** |
| --- | --- | --- | --- | --- | --- |
| 1 | MA | 0 | *Marinomonas sp. 170Z-11* | 99% | JX310217.1 |
| 2 | MA | 0 | *Pseudoalteromonas elyakovii strain BSi20670* | 99% | DQ517879 |
| 3 | MA | 0 | *Pseudoalteromonas sp. R18-19* | 99% | KT449875 |
| 4 | MA | 0 | *Bizionia sp. KJF12-2* | 98% | JQ800199 |
| 7 | MA | 0 | *Pseudoalteromonas sp. H1309/5III* | 99% | LN871567 |
| 8 | MA | 0 | *Pseudoalteromonas sp. ArcN812K13* | 99% | HQ882794 |
| 9 | MO | 7 | *Pseudoalteromonas arctica isolate NSP519* | 99% | FR750943.1 |
| 10 | MO | 7 | *Pseudomonas sp. gap-f-76* | 99% | DQ530477.1 |
| 11 | MO | 7 | *Marinobacter sp. SS7.20* | 99% | KC160651.1 |
| 12 | MO | 7 | *Thalassospira sp. EM202* | 99% | KC469107.1 |
| 16 | MO | 7 | *Pseudomonas sp. gap-f-76* | 99% | DQ530477.1 |
| 19 | DIES | 7 | *Marinobacter sp. HGLP-15* | 99% | KX001828.1 |
| 20 | DIES | 7 | *Thalassospira mesophila strain MBE74* | 99% | NR_114387.1 |
| 23 | DIES | 7 | *Marinomonas sp. 170Z-11* | 99% | JX310217.1 |
| 24 | DIES | 7 | *Bizionia fulviae strain EM7* | 99% | NR_137258.1 |
| 25 | DIES | 7 | *Marinobacter sp. HGLP-15* | 99% | KX001828.1 |
| 26 | DIES | 7 | *Sulfitobacter sp. strain 70415* | 100% | KX889980.1 |
| 27 | DIES | 7 | *Pseudoalteromonas elyakovii strain BSi20670* | 99% | DQ517879.1 |
| 28 | MO | 21 | *Marinobacter sp. SS7.20* | 99% | KC160651.1 |
| 29 | MO | 21 | *Marinobacter sp. HGLP-15* | 99% | KX001828.1 |
| 31 | MO | 21 | *Marinobacter sp. SS7.20* | 99% | KC160651.1 |
| 32 | MO | 21 | *Marinobacter sp. HGLP-15* | 99% | KX001828.1 |
| 34 | MO | 21 | *Pseudoalteromonas elyakovii strain BSi20670* | 99% | DQ517879.1 |
| 36 | DIES | 21 | *Marinobacter sp. HGLP-15* | 99% | KX001828.1 |
| 37 | DIES | 21 | *Marinobacter sp. SS7.20* | 99% | KC160651.1 |
| 38 | DIES | 21 | *Pseudoalteromonas sp. H1309/5III* | 99% | LN871567.1 |
| 39 | DIES | 21 | *Marinobacter sp. HGLP-15* | 99% | KX001828.1 |
| 40 | MO | 42 | *Pseudoalteromonas sp. H1309/5III* | 99% | LN871567.1 |
| 41 | MO | 42 | *Pseudomonas sp. gap-f-76* | 99% | DQ530477.1 |
| 44 | MO | 42 | *Marinobacter sp. HGLP-15* | 99% | KX001828.1 |
| 46 | MO | 42 | *Thalassospira sp. EM202* | 99% | KC469107.1 |
| 48 | MO | 42 | *Pseudoalteromonas sp. ArcN03303* | 99% | HQ882795.1 |
| 49 | MO | 42 | *Marinobacter sp. HGLP-15* | 99% | KX001828.1 |
| 53 | DIES | 42 | *Marinobacter sp. HGLP-15* | 99% | KX001828.1 |

Supplementary Table 7: Specific growth rates (µ) of selected FSC1000 isolates in marine broth based on bacterial optical density measurements (600 nm).

| **Isolate ID** | **Closest relative (BLASTn)** | **µ (h^-1^)** |
| --- | --- | --- |
| 23 | *Marinomonas* sp. 170Z-11 | 0.63 |
| 24 | *Bizionia* *fulviae* strain EM7 | 1.08 |
| 26 | *Sulfitobacter* sp. strain 70415 | 1.17 |
| 27 | *Pseudoalteromonas elyakovii* strain BSi20670 | 0.56 |
| 38 | *Pseudoalteromonas* sp. H1309/5III | 0.93 |
| 44 | *Marinobacter* sp. HGLP-15 | 0.54 |
| 46 | *Thalassospira* sp. EM202 | 0.25 |
| 48 | *Pseudoalteromonas* sp. ArcN03303 | 0.57 |

Supplementary Info 1. Degradation analysis of variance model and significance of variables.

Model Formula:

$$\text{Percent Loss} \sim\text{Oil Type} + \text{Treatment} + \text{Day} + \text{Station} + \text{Oil Type}\times\text{Treatment} + \text{Oil Type}\times\text{Day} + \text{Oil Type}\times\text{Station} + \text{Treatment}\times\text{Day} + \text{Treatment}\times\text{Station} + \text{Day}\times\text{Station} + \text{Oil Type×Treatment}\times\text{Station}$$

Output:

|  | Df | Sum of Sq | Mean of Sq | F Value | Pr(>F) |  |
| --- | --- | --- | --- | --- | --- | --- |
| Oil_Type | 1 | 12 | 12 | 0.527 | 0.4701 |  |
| Treatment | 1 | 15394 | 15394 | 705.026 | 2.00E-16 | *** |
| Day | 3 | 6237 | 2079 | 95.216 | 2.00E-16 | *** |
| Station | 1 | 1138 | 1138 | 52.139 | 3.36E-10 | *** |
| Oil Type × Treatment | 1 | 3 | 3 | 0.138 | 0.71083 |  |
| Oil Type × Day | 3 | 72 | 24 | 1.103 | 0.3533 |  |
| Oil Type × Station | 1 | 110 | 110 | 5.027 | 0.02787 | * |
| Treatment × Day | 3 | 5351 | 1784 | 81.685 | 2.00E-16 | *** |
| Treatment × Station | 1 | 510 | 510 | 23.345 | 6.88E-06 | *** |
| Day ×Station | 3 | 139 | 46 | 2.12 | 0.1047 |  |
| Oil Type × Treatment × Station | 1 | 189 | 189 | 8.647 | 0.00434 | ** |
| Residuals | 76 | 1659 | 22 |  |  |  |

**Legend**

Df – Degrees of Freedom

Sum of Sq – Sums of Squares

Mean of Sq – Mean of Squares

F Value – F Statistic value

Pr(>F) – P value

Differences with p-values <0.1, <0.05, <0.01 and <0.001 are indicated with “∙”, “*”, “**” and “***”, respectively.

Supplementary Info 2. Basal respiration analysis of variance model and significance of

variables

Model Formula:

$$\sqrt{\text{Basal Respiration}} \sim\text{Oil Type} + \text{Day} + \text{Station} + \text{Oil Type}\times\text{Day} + \text{Day}\times\text{Station}$$

Output:

|  | Df | Sum of Sq | Mean of Sq | F value | Pr(>F) |  |
| --- | --- | --- | --- | --- | --- | --- |
| Oil Type | 1 | 0.000468 | 0.000468 | 4.055 | 0.05155 | ∙ |
| Day | 3 | 0.030914 | 0.010305 | 89.284 | < 2e-16 | *** |
| Station | 1 | 0.001838 | 0.001838 | 15.925 | 0.00031 | *** |
| Oil Type × Day | 3 | 0.001432 | 0.000477 | 4.135 | 0.01284 | * |
| Day × Station | 3 | 0.001559 | 0.00052 | 4.503 | 0.00879 | ** |
| Residuals | 36 | 0.004155 | 0.000115 |  |  |  |

**Legend**

Df – Degrees of Freedom

Sum of Sq – Sums of Squares

Mean of Sq – Mean of Squares

F Value – F Statistic value

Pr(>F) – P value

Differences with p-values <0.1, <0.05, <0.01 and <0.001 are indicated with “∙”, “*”, “**” and “***”, respectively.

Supplementary Info 3. Comparison of FSC135 station DGGE matrix with environmental

variables by PerMANOVA analysis

Model Formula:

$$\text{Distance matrix} \sim\text{Day} \times\text{Treatment} (\text{permutations} = 999)$$

Terms added sequentially (first to last).

Output:

|  | Df | Sums of Sqs | Mean of Sqs | F Value | R^2^ | Pr(>F) |  |
| --- | --- | --- | --- | --- | --- | --- | --- |
| Day | 3 | 1.7163 | 0.57211 | 30.083 | 0.53987 | 0.001 | *** |
| Treatment | 1 | 1.0069 | 1.00687 | 52.944 | 0.31671 | 0.001 | *** |
| Day × Treatment | 2 | 0.1897 | 0.09484 | 4.987 | 0.05967 | 0.003 | ** |
| Residuals | 14 | 0.2662 | 0.01902 |  | 0.08375 |  |  |
| Total | 20 | 3.1791 |  |  | 1 |  |  |

**Legend**

Df – Degrees of Freedom

Sum of Sq – Sums of Squares

Mean of Sq – Mean of Squares

F Value – F Statistic value

Pr(>F) – P value

Differences with p-values <0.1, <0.05, <0.01 and <0.001 are indicated with “∙”, “*”, “**” and “***”, respectively.

Supplementary Info 4. Comparison of FSC1000 station DGGE matrix with environmental

variables by PerMANOVA analysis

Model Formula:

$$\text{Distance matrix} \sim\text{Day }\times\text{Treatment} (\text{permutations}=999)$$

Terms added sequentially (first to last).

Output:

|  | Df | Sums of Sqs | Mean of Sqs | F Model | R^2^ | Pr(>F) |  |
| --- | --- | --- | --- | --- | --- | --- | --- |
| Day | 3 | 2.5341 | 0.84470 | 81.672 | 0.43899 | 0.001 | *** |
| Treatment | 1 | 2.7558 | 2.75578 | 266.452 | 0.47739 | 0.001 | *** |
| Day × Treatment | 2 | 0.3379 | 0.16897 | 16.337 | 0.05854 | 0.001 | *** |
| Residuals | 14 | 0.1448 | 0.01034 |  | 0.02508 |  |  |
| Total | 20 | 5.7726 |  |  | 1 |  |  |

**Legend**

Df – Degrees of Freedom

Sum of Sq – Sums of Squares

Mean of Sq – Mean of Squares

F Value – F Statistic value

Pr(>F) – P value

Signif. codes: Differences with p-values <0.1, <0.05, <0.01 and <0.001 are indicated with “∙”, “*”, “**” and “***”, respectively.

Supplementary Info 5 Illumina microbial community, comparison of variables by PerMANOVA analysis.

Model Formula:

$$\text{Normalised OTU table} \sim\text{Station} \times\text{Day} \times\text{Treatment} (\text{permutations} = 999)$$

Terms added sequentially (first to last).

Output:

|  | Df | Sums of Sqs | Mean of Sqs | F Value | R^2^ | Pr(>F) |  |
| --- | --- | --- | --- | --- | --- | --- | --- |
| Station | 1 | 5.8187 | 5.8187 | 254.353 | 0.47847 | 0.001 | *** |
| Day | 1 | 1.1526 | 1.1526 | 50.383 | 0.09478 | 0.001 | *** |
| Treatment | 2 | 2.1254 | 1.0627 | 46.454 | 0.17477 | 0.001 | *** |
| Station × Day | 1 | 0.3963 | 0.3963 | 17.321 | 0.03258 | 0.001 | *** |
| Station × Treatment | 2 | 1.6784 | 0.8392 | 36.684 | 0.13801 | 0.001 | *** |
| Day × Treatment | 1 | 0.1572 | 0.1572 | 6.870 | 0.01292 | 0.001 | *** |
| Station:Day × Treatment | 1 | 0.1004 | 0.1004 | 4.391 | 0.00826 | 0.009 | ** |
| Residuals | 32 | 0.7320 | 0.0229 |  | 0.0602 |  |  |
| Total | 41 | 12.161 |  |  | 1 |  |  |

**Legend**

Df – Degrees of Freedom

Sum of Sq – Sums of Squares

Mean of Sq – Mean of Squares

F Value – F Statistic value

Pr(>F) – P value

Signif. codes: Differences with p-values <0.1, <0.05, <0.01 and <0.001 are indicated with “∙”, “*”, “**” and “***”, respectively.


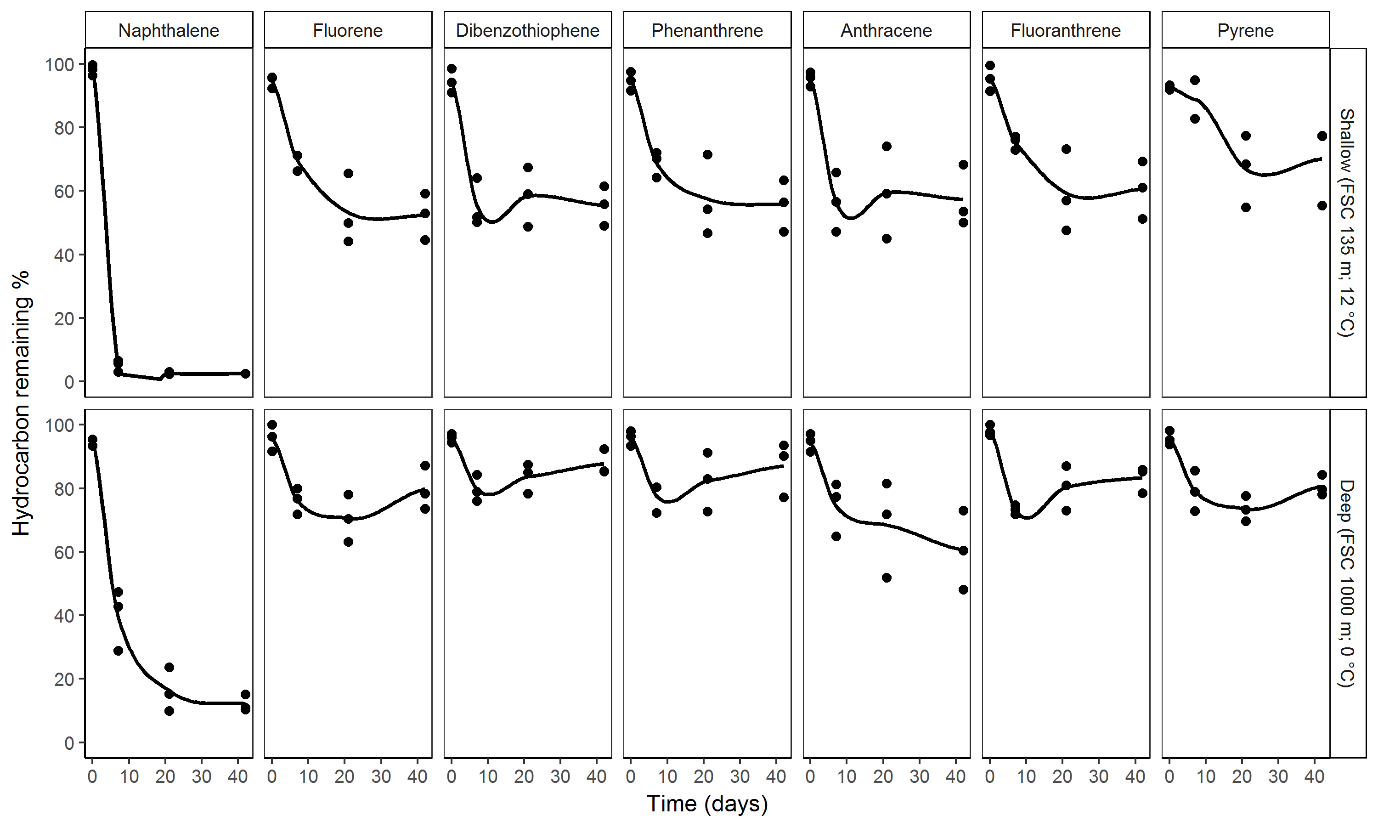

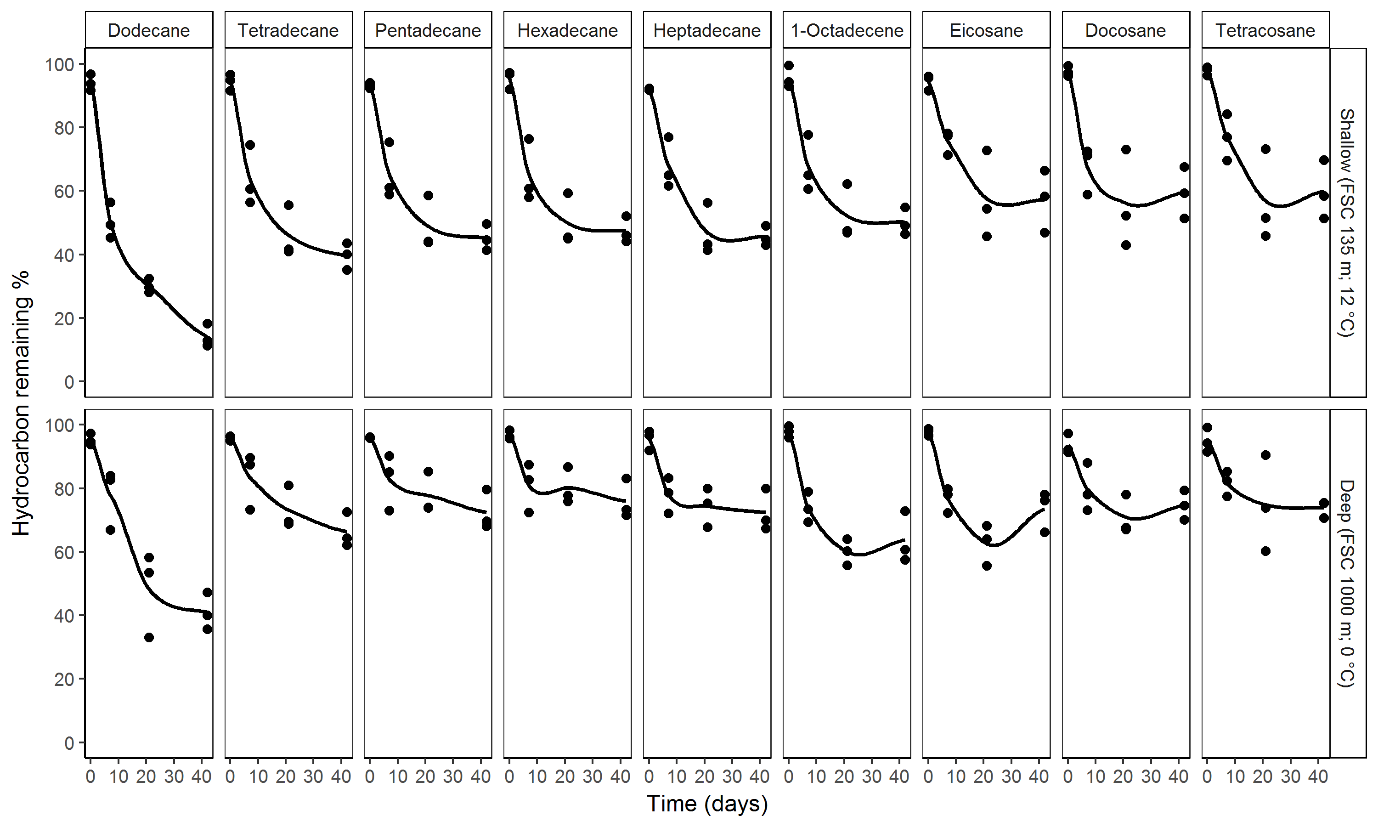


**A**

**B**

Supplementary Figure 1. Degradation of individual A) aliphatic and B) aromatic hydrocarbons in shallow (FSC135, top) and deep (FSC1000, bottom) stations during incubations at 20 °C. Filled circles represent hydrocarbon loss in consortia inoculated treatments and solid lines indicate degradation profiles fitted by locally weighted regression (loess) smoothing. Original station depth and temperature are included on the right y-axis.


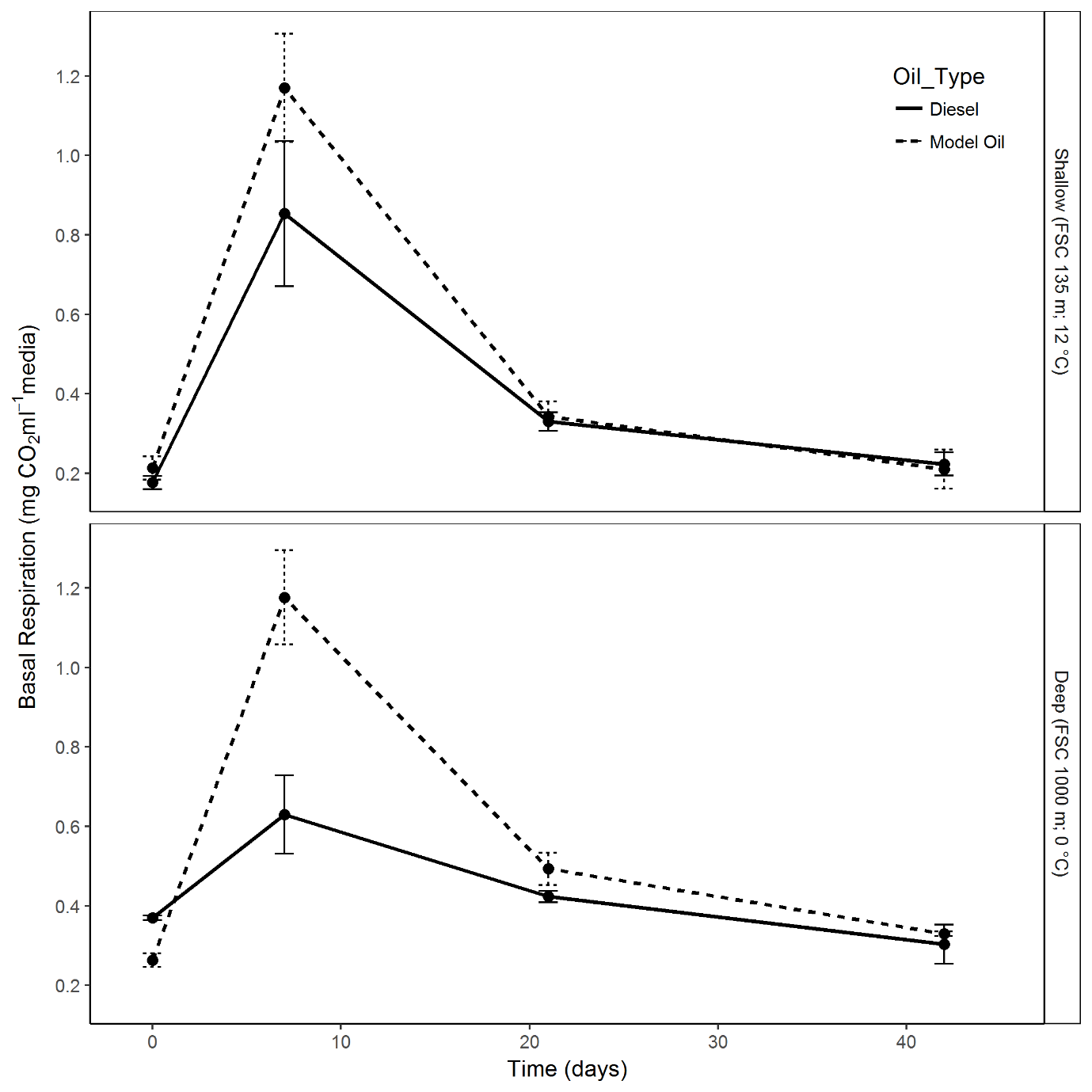


Supplementary Figure 2. Basal respiration of each community on specified sampling time

point over 24 hours. Solid and dashed lines indicate diesel and model oil treatments,

respectively. Original station depth and temperature are included on the right y-axis.

Error bars represent standard error (n = 3).


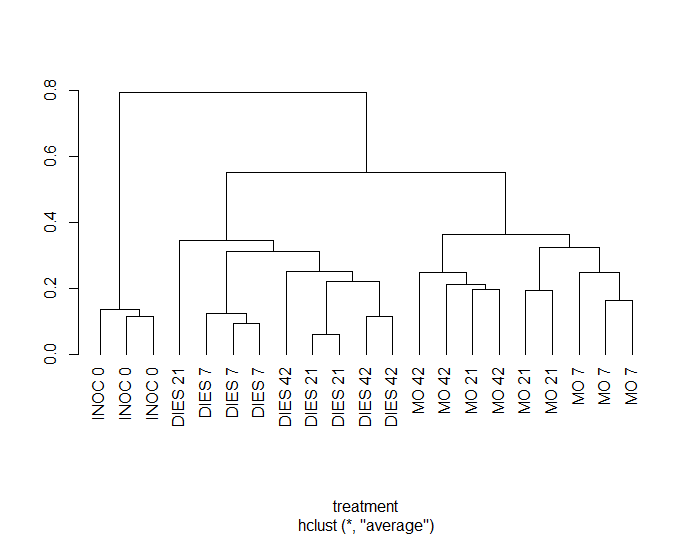

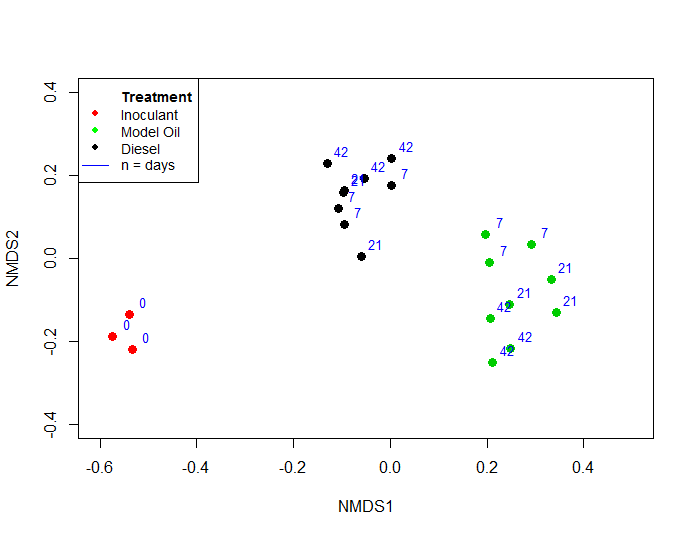


Stress: 0.08

Supplementary Figure 3. FSC135 station DGGE nMDS (Bray-Curtis) plot and cluster analysis (UPGMA).


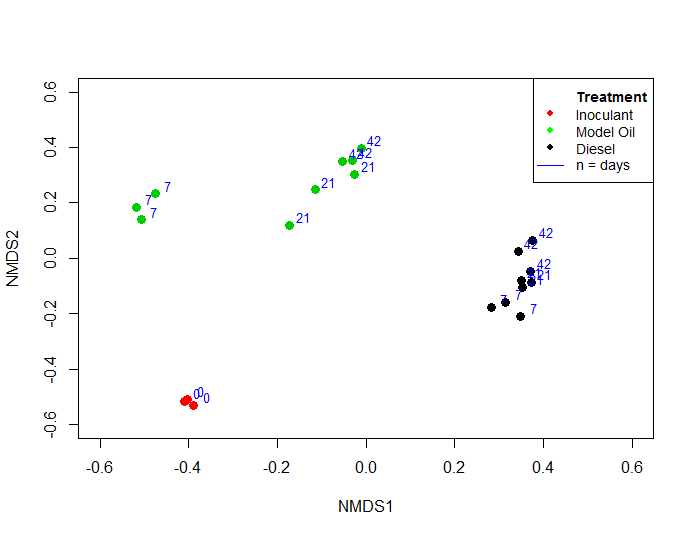

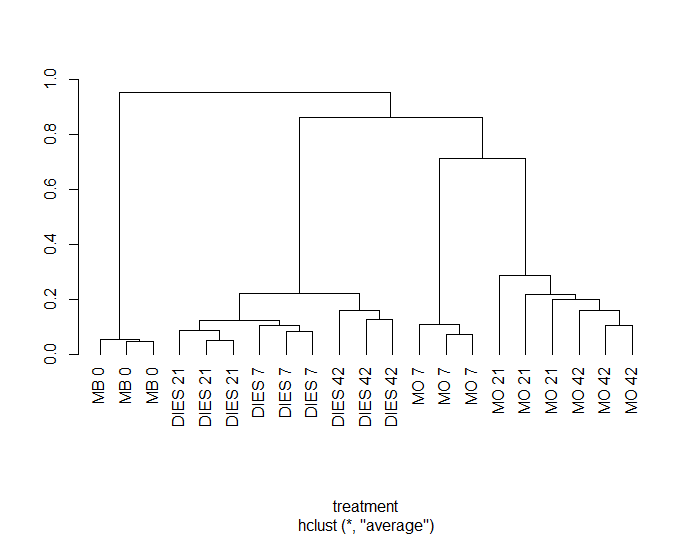


Stress: 0.07

Supplementary Figure 4 FSC1000 station DGGE nMDS (Bray-Curtis) plot and cluster analysis (UPGMA).


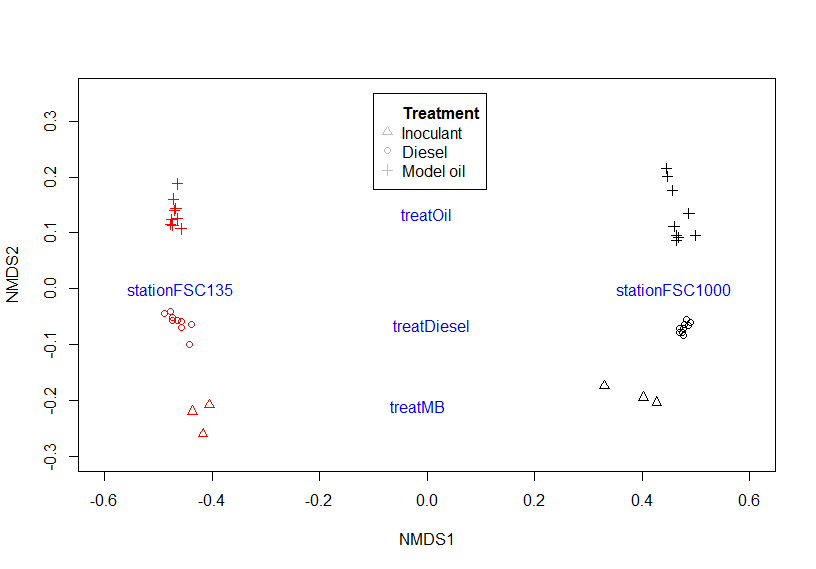


Supplementary Figure 5. Community analysis of both stations and all treatments by nMDS (Bray-Curtis) using Illumina sequencing data output in the form of an OTU table with cut-off at 97% similarity. Labels (station and treatment) represent environmental variables fitted with envfit() function in R.

| **Station** | **Shallow (FSC135 (%))** | | | | | | | **Deep (FSC1000 (%))** | | | | | | |
| --- | --- | --- | --- | --- | --- | --- | --- | --- | --- | --- | --- | --- | --- | --- |
| **Time (days)** | **0** | **7** | | **21** | | **42** | | **0** | **7** | | **21** | | **42** | |
| **Treatment** | **MB** | **MO** | **D** | **MO** | **D** | **MO** | **D** | **MB** | **MO** | **D** | **MO** | **D** | **MO** | **D** |
| *Albirhodobacter* | 12 | 0 | 4 | 0 | 3 | 0 | 4 | 0 | 0 | 0 | 0 | 0 | 0 | 0 |
| *Alcanivorax* | 3 | 24 | 60 | 21 | 70 | 25 | 67 | 0 | 0 | 0 | 0 | 0 | 0 | 0 |
| *Bizionia* | 0 | 0 | 0 | 0 | 0 | 0 | 0 | 22 | 0 | 15 | 0 | 18 | 0 | 16 |
| *Colwellia* | 6 | 0 | 1 | 0 | 0 | 0 | 0 | 0 | 0 | 0 | 0 | 0 | 0 | 0 |
| Unclassified γ-proteobacteria | 0 | 1 | 0 | 1 | 0 | 1 | 0 | 1 | 0 | 35 | 0 | 37 | 1 | 33 |
| *Halomonas* | 19 | 9 | 6 | 9 | 6 | 9 | 6 | 0 | 0 | 0 | 0 | 0 | 0 | 0 |
| *Marinobacter* | 0 | 11 | 0 | 9 | 0 | 9 | 0 | 0 | 5 | 9 | 50 | 14 | 62 | 17 |
| *Marinobacterium* | 0 | 7 | 0 | 7 | 0 | 6 | 0 | 0 | 0 | 0 | 0 | 0 | 0 | 0 |
| *Marinomonas* | 0 | 0 | 0 | 0 | 0 | 0 | 0 | 1 | 4 | 1 | 1 | 2 | 0 | 2 |
| *Oleispira* | 0 | 0 | 0 | 0 | 0 | 0 | 0 | 0 | 5 | 2 | 3 | 0 | 1 | 0 |
| *Pseudoalteromonas* | 46 | 3 | 19 | 2 | 9 | 3 | 8 | 40 | 8 | 12 | 12 | 3 | 4 | 2 |
| *Pseudomonas* | 6 | 40 | 5 | 40 | 5 | 33 | 5 | 27 | 58 | 7 | 20 | 5 | 16 | 5 |
| Unclassified Rhodobacteraceae | 1 | 0 | 1 | 0 | 1 | 0 | 1 | 5 | 1 | 4 | 2 | 5 | 2 | 4 |
| *Thalassospira* | 0 | 3 | 0 | 8 | 2 | 8 | 4 | 2 | 18 | 14 | 12 | 15 | 14 | 18 |

Supplementary Figure 6. Schematic indicating average relative abundance percentages of selected taxa grouped by station, time (days) and treatment. MB indicates marine broth inoculum; MO indicates model oil and D indicates diesel. Colours represent a three-colour scale whereby red indicates 0%, yellow indicates 5% and green indicates >20%, to highlight dominance of certain taxa depending on station, treatment and time.


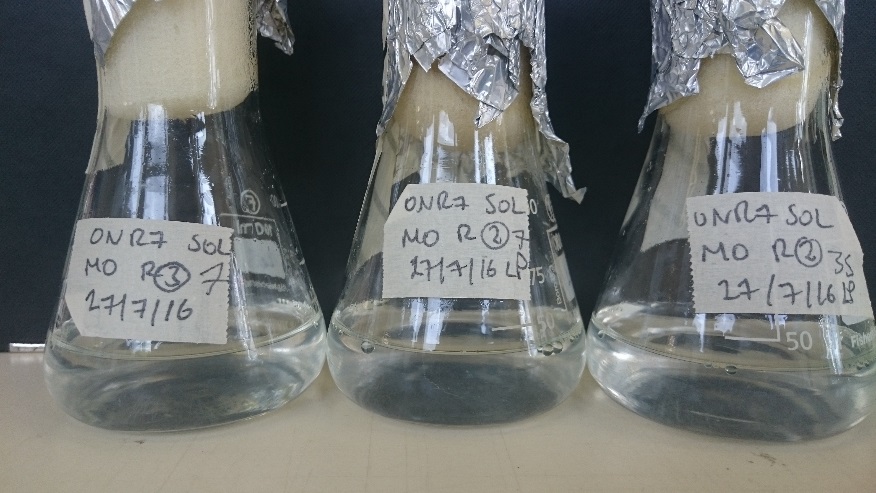

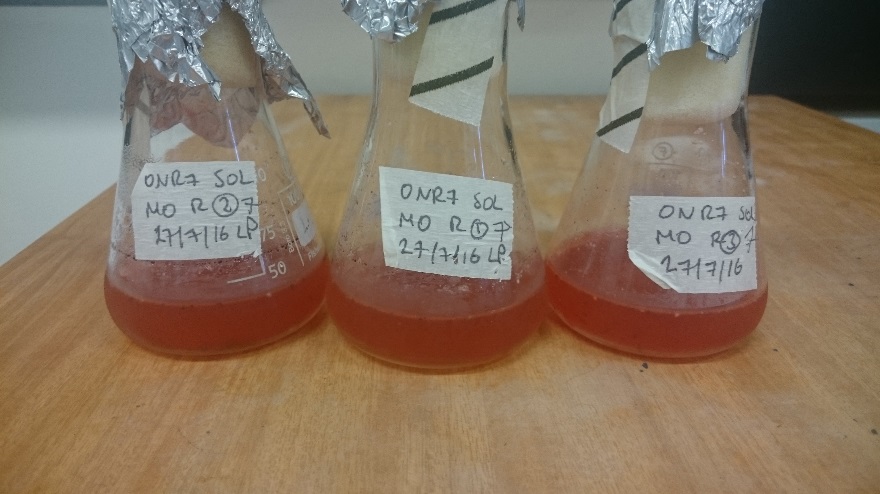

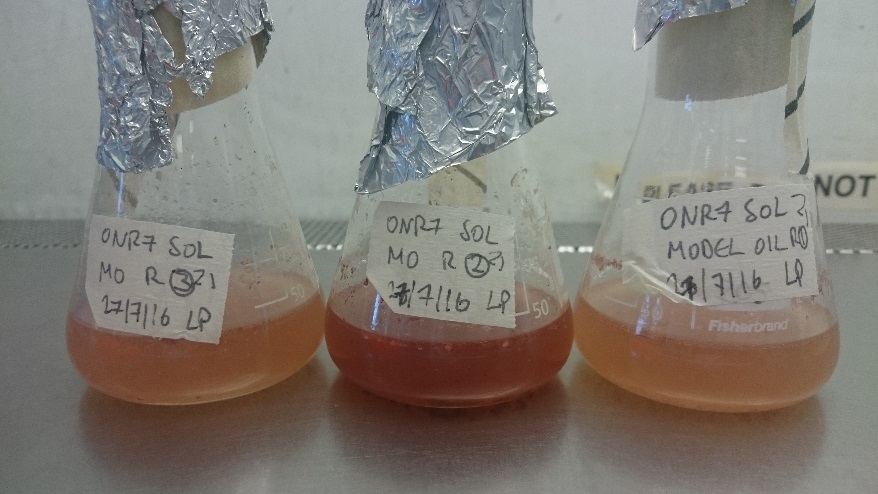


Supplementary Figure 7. Images showing the change in colour of culture medium of FSC135 consortium inoculated with model oil. Increasing turbidity with a colour of reddish-purple is seen from day 0 (top), to day 7 (middle) and to day 21 (bottom).
